# Association between adherence to an antimicrobial stewardship program and mortality among hospitalised cancer patients with febrile neutropaenia: a prospective cohort study

**DOI:** 10.1186/1471-2334-14-286

**Published:** 2014-05-23

**Authors:** Regis G Rosa, Luciano Z Goldani, Rodrigo P dos Santos

**Affiliations:** 1 Postgraduate Program in Medical Sciences of Universidade Federal do Rio Grande do Sul, Porto Alegre, Brazil; 2 Section of Infectious Diseases, Hospital de Clínicas de Porto Alegre, Ramiro Barcelos, 2350, Room 2225, PO Box 90035–903, Porto Alegre, RS, Brazil; 3 Infection Control Committee of Hospital de Clínicas, Porto Alegre, Brazil

**Keywords:** Febrile neutropenia, Antimicrobial agents, Chemotherapy, Program evaluation, Adherence, Mortality

## Abstract

**Background:**

Initial management of chemotherapy-induced febrile neutropaenia (FN) comprises empirical therapy with a broad-spectrum antimicrobial. Currently, there is sufficient evidence to indicate which antibiotic regimen should be administered initially. However, no randomized trial has evaluated whether adherence to an antimicrobial stewardship program (ASP) results in lower rates of mortality in this setting. The present study sought to assess the association between adherence to an ASP and mortality among hospitalised cancer patients with FN.

**Methods:**

We conducted a prospective cohort study in a single tertiary hospital from October 2009 to August 2011. All adult patients who were admitted to the haematology ward with cancer and FN were followed up for 28 days. ASP adherence to the initial antimicrobial prescription was determined. The mortality rates of patients who were treated with antibiotics according to the ASP protocol were compared with those of patients treated with other antibiotic regimens. The multivariate Cox proportional hazards model and propensity score were used to estimate 28-day mortality risk.

**Results:**

A total of 307 FN episodes in 169 subjects were evaluated. The rate of adherence to the ASP was 53%. In a Cox regression analysis, adjusted for propensity scores and other potential confounding factors, ASP adherence was independently associated with lower mortality (hazard ratio, 0.36; 95% confidence interval, 0.14–0.92).

**Conclusions:**

Antimicrobial selection is important for the initial management of patients with FN, and adherence to the ASP, which calls for the rational use of antibiotics, was associated with lower mortality rates in this setting.

## Background

Febrile neutropaenia (FN), secondary to cytotoxic chemotherapy, is a medical emergency that requires the immediate administration of empirical broad-spectrum antimicrobials to prevent the characteristic high levels of morbidity and mortality [1,2]. The signs and symptoms of infection are often subtle or absent because of the lack of an appropriate inflammatory response [3], which underscores the importance of early assessment and management of patients with this syndrome. Patients should be treated initially with empiric intravenous therapy, comprising β-lactam antibiotic monotherapy with antipseudomonal activity (i.e., ceftazidime, cefepime, piperacillin/tazobactam, meropenem or imipenem) [4]. The addition of a second class of antimicrobial to the initial regimen is indicated in special situations, such as the addition of vancomycin in cases of haemodynamic instability, suspected catheter-related infection, pneumonia, or infection of the skin and soft tissue, or the inclusion of metronidazole in cases with gastrointestinal symptoms (e.g., diarrhoea, abdominal pain and perianal pain) [4,5].

The implementation of an antimicrobial stewardship program (ASP) aims to prevent or slow the emergence of multidrug-resistant (MDR) pathogens, reduce the costs related to healthcare and decrease morbidity and mortality [6,7]. Currently, there is a growing interest in the stringent enforcement of ASPs in the context of FN, given the extensive emergence in cancer patients of antimicrobial-resistant strains that are associated with increased risk of morbidity, mortality and cost [8].

Although various studies have demonstrated improvements in clinical outcomes with ASPs (e.g., higher cure rates, lower rates of treatment failure and a lower incidence of infections caused by resistant germs) [9-15], few studies have examined the impact of an ASP on mortality [16]. The available evidence on the effectiveness of ASPs in reducing mortality in FN is scarce [17], and any data originated from studies that were not primarily designed for the determination of this outcome. Thus, the aim of this cohort study was to evaluate the association between adherence to an ASP and mortality among hospitalised cancer patients with FN.

## Methods

### Study design, setting and patients

A prospective cohort study was conducted in the haematology ward of the Hospital de Clínicas de Porto Alegre, Rio Grande do Sul, a teaching hospital and tertiary referral centre for bone marrow transplantation in southern Brazil. In this hospital, the application of an ASP protocol for FN (which is based on the guidelines of the Infectious Diseases Society of America [18]) has been part of the institutional recommendations since 2003. We screened all consecutive cancer patients admitted between October 2009 and August 2011. Patients aged ≥ 18 years with neutropaenia (absolute neutrophil count < 500 cells/mm^3^ or < 1,000 cells/mm^3^ with an expectation of decreasing to < 500 cells/mm^3^ during the following 48 h) and fever (a single axillary temperature measurement ≥ 38.5°C or body temperature ≥ 38.0°C sustained over a 1-h period) were eligible for this study. We excluded subjects who were receiving non-curative cancer treatment, had an indication for outpatient treatment, had a history of allergies to any antibiotics recommended by the ASP of the institution or had neutropaenia with a specific aetiology other than an adverse reaction to chemotherapy.

### Study groups

**Figure 1:**
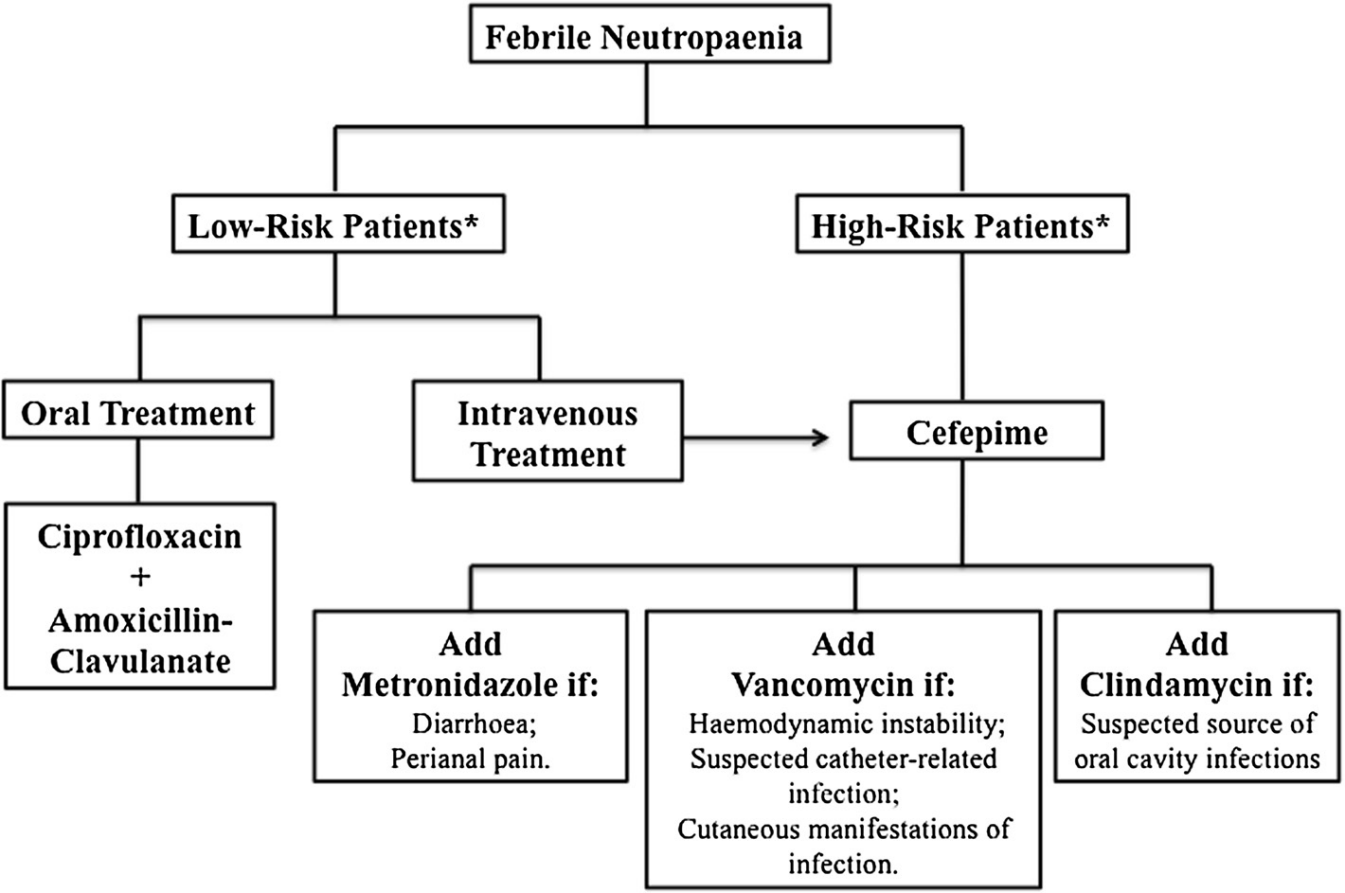
**Initial antimicrobial selection for the in-patient treatment of FN according to the ASP of the Hospital de Clínicas de Porto Alegre.** *High-risk patients = MASCC score < 21 points; Low-risk patients = MASCC score ≥ 21 points. Low-risk patients were treated with intravenous antibiotics if they had one or more of the following: presence of clinical comorbidities, FN after high-dose chemotherapy, expectation of duration of neutropenia > 7 days, documented infection, clinical instability (e.g. hypotension, acute respiratory failure, acute renal failure) and gastrointestinal intolerance (e.g. severe mucositis, vomiting). ASP, antimicrobial stewardship program; FN, febrile neutropaenia; MASCC, Multinational Association for Supportive Care in Cancer.

The main independent variable of the present study was adherence to the hospital’s ASP guidelines [19], which was measured by determining whether the recommended antimicrobial therapy was administered as soon as the symptoms of FN matched those in the ASP guidelines (Figure 1). ASP adherence was determined by a medical research team not associated with patient care at the time of first antibiotic prescribing for a patient with FN. The following situations were classified as non-adherence to the ASP: administration of any antibiotic that was not advised by the ASP; prescription of metronidazole, vancomycin or clindamycin that was not approved by the ASP; and the absence of cefepime in any circumstance, of metronidazole in subjects with diarrhoea or perineal pain, of clindamycin in patients with a suspected oral cavity infection, or of vancomycin during hypotension, suspected catheter-related infection or cutaneous manifestations of infection.

### Covariate definitions

All baseline characteristics were verified at the onset of fever by a medical research team not associated with patient care. The Multinational Association for Supportive Care in Cancer (MASCC) risk index score [20] was applied to determine the risk of serious complications during FN; episodes were classified as high risk if the score was < 21 points and as low risk if the score was ≥ 21 points. Clinical comorbidity was defined as the presence of heart failure, diabetes mellitus, chronic pulmonary disease, chronic liver disease or chronic renal failure. The patients were divided into two groups based on their chemotherapy regimens: a high-dose chemotherapy group that included patients who underwent haematopoietic stem cell transplantation or induction chemotherapy, and a standard-dose chemotherapy group that included patients who underwent consolidation or maintenance chemotherapy. Hypotension was characterised by systolic blood pressure < 90 mmHg. A suspected source of infection in the oral cavity was determined by the presence of a septic tooth or abscess in the mouth. Cutaneous manifestations of infection were defined by the presence of skin signs of inflammation (heat, redness or swelling). A suspected catheter-related infection was defined by the presence of any phlogistic sign at the central venous catheter insertion site. Diarrhoea was characterised by the presence of loose or watery stool. Microbiological studies were performed at the onset of fever according to practice standards, and included two separate blood samples from two different sites. In the absence of an indwelling central venous catheter, the two blood sets were obtained from two distinct peripheral veins. When an indwelling central venous catheter was present, one sample for blood culture was obtained through the indwelling central venous catheter, and the other was collected from a peripheral vein. Bacteraemia by coagulase-negative *Staphylococcus* spp. was defined as two positive results from two independent cultures. Bacteraemia in one positive culture was considered diagnostic for other microorganisms. Polymicrobial bloodstream infection (BSI) was characterised as a bacteraemic episode caused by at least two different pathogens isolated from the same blood sample. The susceptibilities of the isolated pathogens to antibiotics were evaluated according to the recommendations of the Clinical and Laboratory Standards Institute [21]. Multidrug resistant (MDR) bacteraemia was defined as a BSI with methicillin-resistant staphylococci or vancomycin-resistant enterococci for Gram-positive bacteria or as resistance to three or more classes of antimicrobial agents for Gram-negative bacteria.

### Outcome and follow-up

The primary outcome of the study was mortality 28 days after the onset of FN. Patients were followed up through interviews and medical record reviews using a standardised case report form by researchers who were not associated with the attending physician’s team. Follow-up was maintained for 28 days after the onset of fever in FN patients. For patients who were discharged before 28 days, follow-up phone calls were made on the 28th day after the onset of FN to determine whether they were still alive; if a patient was deceased at the time of the phone call, the survival time was calculated based on the date of death reported by the family. A second episode of FN in the same patient was considered only if the patient remained free of signs or symptoms of infection for at least 7 days after the completion of the treatment for the first episode and if all causative organisms were eradicated.

### Statistical analysis

Observational studies are often limited by an imbalance in both known and unknown confounders, which make some FN patients more likely to receive antimicrobial treatment according to ASP guidelines than to receive other antimicrobial treatments. Therefore, we applied a propensity score adjustment to balance baseline characteristics and to reduce the probability of treatment selection bias. The propensity score (probability of receiving an antibiotic recommended by the ASP) was calculated using a stepwise multivariate logistic regression model, in which the dependent variable was treatment according to ASP guideline; all variables that were considered to potentially influence antimicrobial prescribing and that had a *P* value < 0.20 in a univariate analysis were included. In the multivariate model, independent variables were eliminated from the highest to the lowest *P* value but remained in the model if the *P* value was < 0.05 (backward method). The validity of the model was assessed by estimating the area under the receiver operating characteristics (ROC) curve using c-statistics. The balance in the covariates across the study groups was demonstrated by testing for differences in individual covariates between the ASP group and non-ASP group after stratifying by quintiles of propensity scores. The multivariate Cox proportional hazard model adjusted by propensity score was used to assess the association of ASP adherence with 28-day mortality, and all covariates with a *P*-value < 0.15 in a univariate analysis were included. In the multivariate model, independent variables were eliminated according to backward selection but remained in the model if the *P*-value was < 0.05. The hazard ratios (HRs) were estimated along with the 95% confidence intervals (CIs). Propensity score weighted Kaplan–Meier curves were used to calculate the time-dependent occurrence of death; the log-rank test was used for comparisons between groups. The software used for the statistical analysis was STATA version 12 (StataCorp LP, TX, USA).

### Ethical issues

Written, informed consent was obtained from all study participants. The institutional review board of the Hospital de Clínicas de Porto Alegre approved the study.

## Results

### Study population

In total, twenty cases (11 in the ASP group and 9 in the non-ASP group) met criteria for exclusion from the cohort. The reasons for exclusion were indication for outpatient treatment (9 cases in the ASP group and 7 cases in the non-ASP group), non-curative cancer treatment (1 case in the ASP group and 1 case in the non-ASP group), history of allergies to any antibiotics recommended by the ASP (1 case in the non-ASP group) and aetiology of FN other than an adverse reaction to chemotherapy (1 case in the ASP group). Overall, 307 episodes of FN (in 169 patients) were evaluated during the study period. Of these, 81% occurred after 48 h of hospitalisation (250 cases). Seventy-one subjects (42% of the study population) had two or more episodes of FN, and the maximum number of episodes studied per patient was four. Antibiotic prophylaxis with fluoroquinolones was not administered to any patient. There were no losses to follow-up. The overall rate of adherence to the ASP of this institution was 53% (162 cases). The mortality of this cohort was 9.4% (29 patients), although no deaths occurred during the follow-up periods of two or more FN episodes in the same patient.

The baseline clinical characteristics of all episodes of FN evaluated in the present cohort are shown in Table 1. As a result of the non-randomized design, the baseline characteristics of patients with FN receiving antimicrobial therapy recommended by the ASP were different from those of patients receiving antimicrobial therapy non-adherent to the ASP. These differences were particularly important regarding the presence of clinical comorbidity; history of antibiotic use in the previous 30 days; proportion of FN episodes with high-risk MASCC scores; and hypotension, diarrhoea, perianal pain and cutaneous manifestations of infection at the time of diagnosis of FN. However, after weighting by the propensity score, all differences decreased to non-significant values, which suggested that propensity score matching adjusted appropriately for the initial treatment selection bias (Table 1).

**Table 1:** Comparison of baseline variables between FN cases receiving antimicrobial therapy recommended by the ASP and those receiving other antimicrobial regimens (non-ASP)

**Variables**	**ASP group N = 162**	**Non-ASP group N = 145**	**PS adjusted, *P *-value**
Age, mean ± SD, years	41.8 ± 14.5	39.4 ± 13.8	0.07
Female sex (%)	80 (49.4)	68 (46.9)	0.76
Clinical comorbidity (%)	33 (20.4)	43 (29.6)	0.31
Type of neoplastic disease			0.71
- Haematological (%)	128 (79.0)	114 (78.6)	
- Solid tumour (%)	34 (21.0)	31 (21.4)	
Chemotherapy regimens			0.95
- High-dose (%)	88 (54.3)	76 (52.4)	
- Standard-dose (%)	74 (45.7)	69 (47.6)	
Relapsing underlying disease status (%)	77 (47.5)	78 (53.8)	0.30
Antibiotic use in the previous 30 days (%)	49 (30.2)	61 (42.0)	0.15
Hospitalisation in the previous 30 days (%)	59 (36.4)	61 (42.0)	0.59
Median ANC at the time of diagnosis of FN (IQR), cells/mm^3^	175 (60–340)	100 (40–260)	0.61
ANC < 100 cells/mm^3^ at the time of FN (%)	62 (38.3)	68 (46.9)	0.78
Nosocomial-acquired FN (%)	127 (78.4)	123 (84.8)	0.83
Hypotension at the time of diagnosis of FN (%)	4 (2.5)	27 (18.6)	0.46
Diarrhoea at the time of diagnosis of FN (%)	14 (8.6)	48 (33.1)	0.10
Perianal pain at the time of diagnosis of FN (%)	6 (3.7)	26 (17.9)	0.67
Suspected source of oral cavity infections (%)	3 (1.8)	12 (8.3)	0.85
Cutaneous manifestations of infection (%)	8 (5.0)	42 (28.9)	0.92
High-risk MASCC score (%)	35 (21.6)	48 (33.1)	0.95

**Note.***P*-values are provided for the differences between the two groups after propensity score adjustment.

PS, propensity score; SD, standard deviation; ANC, absolute neutrophil count; IQR, interquartile range (P25–P75).

The microbiological characteristics of all FN cases with documented BSI are shown in Table 2. During the study period, 115 BSIs were diagnosed. The predominant blood isolates were *Escherichia coli* (42%), coagulase-negative staphylococci (31%), *Klebsiella pneumonia* (11%), *Pseudomonas aeruginosa* (9%), viridans streptococci (7%) and *Enterococcus* spp. (3%). Among all BSIs evaluated, 38 episodes (33%) were caused by MDR bacteria; of these, 68% were caused by Gram-positive bacteria, 29% were caused by Gram-negative bacteria, and 3% were caused by both Gram-positive and Gram-negative bacteria.

**Table 2:** Microbiological characteristics of FN cases

**Variables**	**ASP group**	**Non-ASP group**
	**N = 162**	**N = 145**
Documented BSI (%)	57 (35.2)	58 (40.0)
Blood isolates		
**-***Escherichia coli* (%)	20 (12.3)	24 (16.5)
*-* Coagulase-negative staphylococci (%)	16 (9.9)	20 (13.7)
**-***Klebsiella pneumonia* (%)	6 (3.7)	7 (4.8)
*- Pseudomonas aeruginosa* (%)	6 (3.7)	5 (3.4)
*-* Viridans strepcococci (%)	6 (3.7)	2 (1.3)
**-***Enterococcus* spp. (%)	2 (1.2)	2 (1.3)
**-***Serratia* spp. (%)	1 (0.6)	1 (0.7)
**-***Enterobacter* spp. (%)	2 (1.2)	0 (0)
**-***Candida* spp. (%)	0 (0)	2 (1.3)
**-***Salmonella* spp. (%)	1 (0.6)	0 (0)
**-***Staphylococcus aureus* (%)	0 (0)	1 (0.7)
**-***Kocuria varians* (%)	1 (0.6)	0 (0)
BSI involving Gram-positive MDR bacteria	12 (7.4)	15 (10.3)
BSI involving Gram-negative MDR bacteria	9 (5.5)	3 (2.0)

**Note:** There were 12 cases of polymicrobial BSI (5 in the ASP group and 7 in the non-ASP group).

BSI, bloodstream infection; MDR, multidrug resistant.

The proportion of FN episodes with time to initial antibiotic administration > 1 h was 25.3% in the ASP group and 24.8% in the non-ASP group. The overall *in vitro* sensitivity rate of blood isolates to the initial antibiotic regime administered was 88% in the ASP group and 86% in the non-ASP group. No case was eligible for oral treatment at the onset of FN, mainly due to the high prevalence of clinical comorbidities, FN after high-dose chemotherapy and expectation of duration of neutropenia > 7 days among cases stratified as low risk by MASCC score. Additionally, no case with an incorrect antibiotic dose was documented in the cohort.

### Antimicrobial regimens prescribed with no adherence to the ASP

Antimicrobial monotherapy was more frequently prescribed to FN patients when there was no adherence to the ASP (Table 3). Among the monotherapies, cefepime was the most commonly prescribed, followed by piperacillin/tazobactam. In cases prescribed combination therapy, the most frequently used were cefepime + metronidazole and cefepime + vancomycin. In cases of non-adherence to the ASP, the prescribed antimicrobial spectrum was considered overtreatment in 11% of cases (16 episodes), and insufficient in 89% (129 episodes) according ASP guideline. In the insufficient therapy group, there were indications for the use of vancomycin, metronidazole, clindamycin and cefepime in 56% (73 episodes), 51% (66 episodes), 5% (7 episodes) and 2% (3 episodes) of cases, respectively.

**Table 3:** Antimicrobial regimens prescribed to 145 non-ASP cases

**Antibiotic scheme**	**Number (%)**
**Monotherapy**	129 (89.0)
- Cefepime	95 (65.6)
- Piperacillin/tazobactam	26 (17.9)
- Imipenem/meropenem	7 (4.8)
- Ciprofloxacin	1 (0.7)
**Combination therapy**	16 (11.0)
- Cefepime + metronidazole	5 (3.4)
- Cefepime + vancomycin	3 (2.0)
- Cefepime + amikacin	1 (0.7)
- Cefepime + azithromycin	1 (0.7)
- Cefepime + vancomycin + ampicillin	1 (0.7)
- Piperacillin/tazobactam + vancomycin	3 (2.1)
- Cefuroxime + azithromycin	1 (0.7)
- Vancomycin + metronidazole	1 (0.7)

### Predictors of initial antimicrobial prescribing according to the ASP

We found several clinical factors that were significantly associated with antibiotic treatment according to ASP recommendations (Table 4). Older patients were more likely to be treated according to the ASP. Conversely, hypotension, diarrhoea, perianal pain and cutaneous manifestations of infection at the time of diagnosis of FN, and the presence of a comorbidity were more frequently found in cases with treatment non-adherent to ASP guidelines. The propensity score model showed an excellent discriminatory ability (area under the ROC curve = 89%). Microbiological characteristics were not entered into the propensity score modeling because its results were unknown at the baseline.

**Table 4:** Multivarate logistic model of baseline factors significantly associated with antimicrobial treatment according ASP guideline

**Variables**	**Odds ratio**	**95% CI**	***P*-value**
Age, years	1.03	1.007–1.06	0.01
Clinical comorbidity	0.40	0.18–0.89	0.02
Hypotension at the time of diagnosis of FN	0.04	0.01–0.15	<0.001
Diarrhoea at the time of diagnosis of FN	0.05	0.02–0.11	<0.001
Perianal pain at the time of diagnosis of FN	0.07	0.02–0.21	<0.001
Suspected source of oral cavity infections	0.05	0.01–0.22	<0.001
Cutaneous manifestations of infection	0.04	0.01–0.11	<0.001

**Note:** Variables entered into the model: age, clinical comorbidity, ANC at the time of the diagnosis of FN, nosocomial-acquired FN, antibiotic use in the previous 30 days, high-risk MASCC score, hypotension at the time of diagnosis of FN, diarrhoea at the time of diagnosis of FN, perianal pain at the time of diagnosis of FN, suspected source of oral cavity infections, and cutaneous manifestations of infection.

CI, confidence interval.

### Mortality

**Figure 2:**
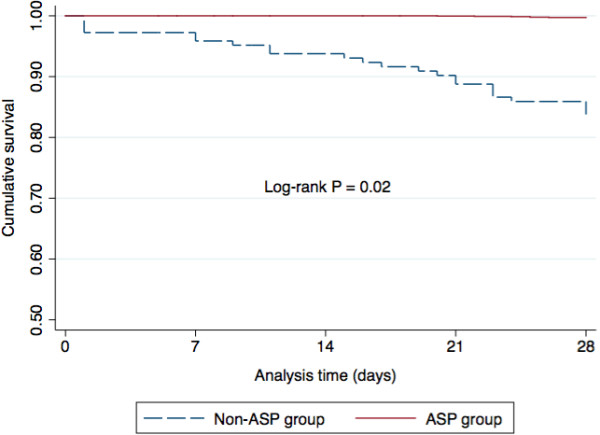
Kaplan–Meier curves of 28-day mortality according adherence to ASP after propensity score weighting.

The 28-day mortality rate was significantly lower in patients who were treated according to the ASP recommendations compared with the non-ASP group (log-rank *P* = 0.02) (Figure 2).

In the univariate Cox proportional hazards model, 28-day mortality during FN (Table 5) was associated with non-adherence to the ASP recommendations (*P* = 0.001), relapsing disease stages (*P* = 0.001), a confirmed BSI (*P* = 0.001), a high-risk MASCC score (*P* < 0.001), hypotension at the time of diagnosis of FN (*P* < 0.001) and time to antibiotic therapy > 1 h (*P* = 0.001). After the propensity score-weighted multivariate analysis (Table 6), the variables that constituted independent predictors for mortality included presentation with relapsing disease stages (*P* = 0.003), a confirmed BSI (*P* = 0.003), a high-risk MASCC score (*P* = 0.008), and time to antibiotic therapy > 1 h (*P* = 0.001). Adherence to the ASP was independently associated with a higher survival rate (*P* = 0.03). Mortality was attributable to infection in all 29 patients who died.

**Table 5:** Prognostic factors of 28-day mortality in FN patients by Cox regression univariate analysis

**Variables**	**Mortality group**	**Survival group**	**HR (95% CI)**	***P*-value**
	**N = 29**	**N = 278**		
Age, mean ± SD, years	41.6 ± 15.0	40.6 ± 14.1	1.00 (0.98–1.03)	0.62
Clinical comorbidity (%)	6 (20.6)	70 (25.1)	0.82 (0.33–2.01)	0.66
Type of neoplastic disease	24 (82.8)	218 (78.4)	1.20 (0.45–3.15)	0.70
- Haematological (%)	5 (17.2)	60 (21.6)	**–**	**–**
- Solid tumour (%)				
Relapsing underlying disease status (%)	23 (79.3)	132 (47.4)	4.30 (1.75–10.58)	0.001
Chemotherapy regimens	9 (31.0)	155 (55.8)	0.39 (0.17–0.86)	0.02
- High-dose (%)	20 (69.0)	123 (44.2)	–	-
- Standard-dose (%)				
Median ANC at the time of the diagnosis of FN (IQR), cells/mm^3^	130 (40–300)	130 (50–310)	1.00 (0.99–1.00)	0.37
ANC < 100 cells/mm^3^ at the time of the diagnosis of FN	16 (55.1)	114 (41.0)	1.68 (0.80–3.49)	0.16
Duration of neutropaenia, median (IQR), days	8 (4–20)	9 (6–17)	0.97 (0.93–1.01)	0.23
Documented BSI (%)	20 (69.0)	95 (34.2)	3.91 (1.78–8.60)	0.001
BSI involving Gram-positive MDR bacteria (%)	2 (6.9)	25 (9.0)	0.74 (0.17–3.12)	0.68
BSI involving Gram-negative MDR bacteria (%)	3 (10.3)	9 (3.2)	2.70 (0.81–8.94)	0.10
High-risk MASCC score (%)	18 (62.1)	65 (23.4)	5.03 (2.37–10.65)	<0.001
Hypotension at the time of diagnosis of FN (%)	9 (31.0)	22 (7.9)	4.72 (2.14–10.37)	<0.001
Time to antibiotic therapy >1 h	15 (51.7)	62 (22.3)	4.13 (1.85–9.20)	0.001
*In vitro* sensitivity of blood isolates to initial antibiotic treatment administered (%)	22 (75.8)	246 (88.4)	0.47 (0.20–1.10)	0.08
ASP adherence (%)	6 (20.7)	156 (56.1)	0.21 (0.08–0.53)	0.001

HR, hazard ratio; SD, standard deviation; IQR, interquartile range (P25–P75); BSI, bloodstream infection; MDR, multidrug resistant.

**Table 6:** Prognostic factors of 28-day mortality in FN patients by Cox regression multivariate analysis adjusted using a propensity score

**Variable**	**HR (95% CI)**	***P*-value**
Relapsing underlying disease status	4.43 (1.65–11.86)	0.003
Documented BSI	3.78 (1.55–9.20)	0.003
High-risk MASCC score	3.01 (1.33–6.83)	0.008
Time to antibiotic >1 h	3.85 (1.71–8.62)	0.001
ASP adherence	0.36 (0.14–0.92)	0.03

**Note:** Variables entered into the model: propensity score, ASP adherence, time to antibiotic >1 h, *in vitro* sensitivity of blood isolates to initial antibiotic treatment administered, high-risk MASCC score, documented BSI, BSI involving Gram-negative MDR bacteria, relapsing underlying disease status, hypotension at the time of diagnosis of FN, and high-dose chemotherapy regimens.

## Discussion

The present study showed that, after risk adjustment, hospitalised FN cases treated according to ASP recommendations had a relative risk reduction in 28-day mortality of 64% compared with cases receiving antimicrobial treatment non-adherent to ASP recommendations.

Our findings reinforce the relevance of appropriate adherence to ASPs, and the importance of the continued evaluation of these programs for the medical community. ASPs are often based on the best-available evidence, with consideration of local data on antimicrobial resistance, and primarily seek to reduce morbidity and mortality in patients for whom the protocols were designed. Additionally, ASPs are often focused on implementing strategies that prioritize the rational use of antimicrobials to reduce the incidence of antibiotic-resistant bacteria. This is of paramount importance in the current scenario of bacterial multidrug resistance associated with low availability of effective antibiotics to treat these infections.

Previous studies have shown rates of mortality and adherence to an ASP similar to those found in our cohort. For example, Kuderer et al. reported in-hospital mortality of 9.5% in subjects with FN [22], and compliance to an ASP was found in 56% of FN cases in a study by Jin et al. [23]. The impact of ASP implementation on clinical outcomes in different infectious diseases [24,25] serves as a theoretical basis for the hypothesis that ASP reduces mortality.

The relatively low adherence to the ASP found in our study indicates the need for strategies to increase medical adherence to ASPs. We hypothesise that noncompliance with the ASP in our study is related to prescribing practices of multi-tasking attending physicians, who do not take time to consult the institutional ASP protocol. In the present study, most FN cases non-adherent to the ASP were treated with monotherapy even though there was a high prevalence of specific factors that indicated the need for changes in the initial antibiotic regimen (i.e., diarrhoea, perianal pain, hypotension, cutaneous manifestation of infection, and suspected oral cavity infection). Moreover, the prescribed antimicrobial spectrum was classified as insufficient in most cases in the non-ASP group.

The present study had some limitations. Antimicrobial modifications during the course of FN were not examined. Furthermore, the study was susceptible to biases inherent to observational studies (selection, assessment and confounding); however, the possibility of systematic errors was minimised by the proper measurement of variables and outcomes with previously defined objective criteria, the use of standardised data collection and follow-up performed by a research team that was not involved in patient care, and also the application of propensity scores, which allowed the balancing of important covariates in the two study arms.

## Conclusion

This study found that adherence to the ASP was associated with lower mortality. Additional studies are required to assess the impact of ASP adherence on mortality for different nosocomial infections. The investigation of factors leading to non-adherence by prescribers should also be thoroughly explored to develop new approaches to increase the adherence to ASPs.

## Abbreviations

FN: Febrile neutropaenia; ASP: Antimicrobial Stewardship Program; MASCC: The Multinational Association for Supportive Care in Cancer.

## Competing interests

The authors declare that they have no competing interests.

## Authors’ contributions

RGR, RPS and LZG are responsible for the concept, design and implementation of this study. RPS and LZG designed the methods of data collection. RGR coordinated data collection and performed data analysis. All authors read and approved the final manuscript.

## Pre-publication history

The pre-publication history for this paper can be accessed here:

http://www.biomedcentral.com/1471-2334/14/286/prepub
